# Research on the impact and mechanism of financial development on new urbanization: A case study of the Yangtze River Economic Belt

**DOI:** 10.1371/journal.pone.0289758

**Published:** 2023-08-10

**Authors:** Yaobin Liu, Yan Guo, Dejin Xie, Xiaodong Xiao, Weihui Hu

**Affiliations:** 1 School of Economics and Management, Nanchang University, Nanchang, China; 2 School of Finance, Jiangxi Normal University, Nanchang, China; Chengdu University of Information Technology, CHINA

## Abstract

Based on panel data of 108 cities in China’s Yangtze River Economic Belt from 2003 to 2019, a multiple mediation model is used in this study to assess the impact and mechanism of financial development on new urbanization. The main conclusions are that financial development can directly promote the improvement of new urbanization and indirectly improve the level of new urbanization by increasing infrastructure investment, optimizing industrial structure, and enhancing human capital. Further, the financial development of middle-upstream cities has a stronger promoting effect on new urbanization. Whereas the financial development of downstream cities mainly promotes the construction of new urbanization through both infrastructure investment and industrial structure optimization, middle-upstream cities rely more solely on infrastructure investment.

## Introduction

Urbanization is a sustained and strong trend in the 21st century, which is known as a hallmark of economic development [1]. A higher level of per capita income is accompanied by a higher level of urbanization in many developed countries [2,3]. The rapid urbanization of developing countries has promoted global population growth, migration, economic development, and human well-being [4–6]. As one of the cores of modern economic development, finance plays an important role in providing funds and supporting resource allocation, and is an important way to promote the development of urbanization [7]. As a low carbon emissions industry, the financial industry is critical for the sustainable development of cities [8].

As the largest developing country in the world, China’s urbanization has been expanding extensively for a long time since the reform and opening up. The rapid improvement of urbanization in China has also brought about a series of resource and environmental problems [9,10], which will undoubtedly pose greater challenges to the sustainable development of the global city. Therefore, the Chinese government clearly pointed out the necessity of improving the quality of urbanization and promoting the construction of a new type of urbanization centered on people. In recent decades, some cities in China have experienced rapid development in the financial industry and new urbanization [11,12]. Financial development has become the impetus for new urbanization. The Yangtze River Economic Belt is one of the fastest-growing watershed economic zones in the world, and it has the most complete urban system in the world. Therefore, it is of great theoretical and practical significance to explore the impact and mechanism of financial development on new urbanization in promoting its construction. This scenario is not only conducive to the economic and social development of the Yangtze River Economic Belt but also promotes the construction of new urbanization in China, and even serves as a reference for sustainable urbanization in other developing countries.

To examine whether financial development drives urbanization, previous studies have investigated the impact of financial development on urbanization from various perspectives, such as financial scale, financial structure, and financial efficiency. Relevant studies have mainly focused on the positive effects of financial development on urbanization [13–17]. While limited studies have pointed out that financial support has a single and significant threshold effect on land urbanization, finance has an inhibiting effect when it is more than the threshold value [18]. Moreover, few scholars have studied the mechanism of financial development in new urbanization, particularly through quantitative analysis.

This paper builds an analytical framework for the mechanism of financial development on new urbanization, and analysis it with a multiple mediation model by using a sample of 108 cities in the Yangtze River Economic Belt of China from 2003 to 2019. The marginal contributions of this paper are as follows: (1) It constructs an analytical framework of the impact of financial development on new urbanization, thus expanding the field. (2) The multiple intermediary model is used to identify the specific mechanism of financial development in new urbanization, and the regional heterogeneity of the impact is further explored to provide implications for the formulation of differentiated policies in different regions.

## Theoretical analysis and research hypotheses

### The direct effect of financial development on new urbanization

The direct effect of financial development on new urbanization is mainly reflected in the following three aspects: First, banks can provide credit support through retail loans to small and medium-sized enterprises, which promotes their development and creates more employment opportunities to attract population agglomeration, thus promoting population urbanization [19]; Second, unlike tangible products, financial services are mostly intangible, which consume relatively less natural resources and have a relatively low degree of environmental damage. Therefore, financial development can alleviate ecological pressures, such as resource constraints and environmental damage, thus promoting green urbanization [20]; Third, financial development can provide financial support for urban development to promote urban economic growth, thus accelerating economic urbanization [21]. Therefore, the first hypothesis is going to be put forward as follows:

Hypothesis 1: Financial development can promote the construction of new urbanization.

### The indirect effects of financial development on new urbanization

This study holds that financial development can not only directly promote the construction of new urbanization but also indirectly affect new urbanization by accelerating the accumulation of production factors and optimizing the allocation of production factors [7]. From the perspective of production factor accumulation, financial development has an impact on new urbanization by increasing infrastructure investment and improving human capital levels. Further, financial development affects the construction of new urbanization by optimizing the allocation of production factors among industries—that is, optimizing the industrial structure [16]. The specific mechanism is as follows:

The intermediary transmission mechanism of infrastructure investment mainly includes two aspects. On the one hand, infrastructure is characterized by a large investment scale, low profitability, and a long construction cycle. It is difficult to support infrastructure investment solely through financial input, while financial development only provides financial support for infrastructure construction [22]. Therefore, financial development can increase the supply of funds for infrastructure construction, accelerate infrastructure construction, and thus promote the construction of new urbanization. On the other hand, the financial sector, with its advantages in information collection and risk management, can invest limited funds in high-quality projects, which helps optimize the allocation of funds in infrastructure construction, promote infrastructure construction, and ultimately promote the construction of new urbanization [23].

On the level of industrial structure optimization, the financial system can provide financial support for the city’s emerging industries, advantageous industries, sunrise industries, and other key industries, help in their rise and development, and thus promote the continuous optimization of the urban industrial structure. The capital needed for the development of various industries in the urban economy is mainly obtained through bank loans, the issuance of stocks, bonds, and other forms of financing [24]. The profit-driven financial institutions and the price discovery function of the financial market foster the allocation of limited financial resources to industries with fast growth and high return on investment, so that advantageous industries can experience considerable development, thus optimizing the industrial structure [25]. The optimization of industrial structure can promote the development of high-quality industrial clusters in urban areas and improve the employment carrying capacity of urban areas for rural migrant populations, thereby driving population agglomeration toward urban areas and promoting the process of new urbanization [26].

From the perspective of human capital promotion, the financial sector can provide people with loan support for education, housing, employment, and other aspects, which enhances the survival and development ability of urban residents, thus improving the level of human capital [27,28]. Further, the financial sector can improve the attractiveness of urban talents by guiding funds to public services, such as education, pension, medical care, fertility, and culture. The improvement of human capital levels can effectively promote cities to get rid of the extensive urbanization development mode and turn to the path of new urbanization construction [1]. Therefore, the second hypothesis is as follows:

Hypothesis 2: Financial development promotes the construction of new urbanization by increasing infrastructure investment, optimizing industrial structure, and enhancing human capital.

**Fig 1:**
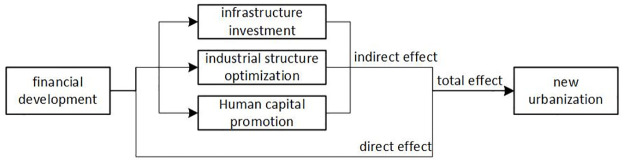
Theoretical framework.

Fig 1 summarizes the theoretical framework constructed in this study.

## Methods and data

### Methods

#### Benchmark model

To examine the impact of financial development on new urbanization, this study constructed the following benchmark regression equation:

$$NU_{it}=\alpha +\beta FD_{it}+\gamma Z_{it}+\mu_{i}+\varepsilon_{it},$$ (1)

where *i* represents the city and *t* represents the year, *NU* represents the level of new urbanization, *FD* represents the level of financial development, *Z* is a series of control variables, including macro-control ability, commercial service activity level, and technology research and development level, *μ*_*i*_ is the fixed effect of cities, and *ε*_*it*_ represents a random perturbation term.

#### Multiple mediation model

Based on the above theoretical analysis, financial development may promote new urbanization through infrastructure investment, industrial structure optimization, and human capital enhancement. To further explore the mechanism of financial development in new urbanization, this study constructed the following multiple mediation model for mechanism identification testing, referring to the multiple mediation model proposed by Hayes [29]:

$$II_{it}=\mu_{i}+\alpha_{1}FD_{it}+\beta_{1}Z_{it}+\varepsilon_{it}$$ (2)

$$ISO_{it}=\mu_{i}+\alpha_{2}FD_{it}+\beta_{2}Z_{it}+\varepsilon_{it}$$ (3)

$$HC_{it}=\mu_{i}+\alpha_{3}FD_{it}+\beta_{3}Z_{it}+\varepsilon_{it}$$ (4)

$$NU_{it}=\mu_{i}+\lambda FD_{it}+\gamma M_{it}+\theta Z_{it}+\varepsilon_{it},$$ (5)

where *II*, *ISO*, and *HC* are the three intermediary variables of infrastructure investment, industrial structure optimization, and human capital level, respectively. *M* represents the three intermediary variables, and the other variables are as described in Eq 1.

### Variable

#### Interpreted variable

New urbanization (*NU*): This study refers to the research of Huang et al. [1] and constructs a comprehensive evaluation index system for new urbanization from five aspects: social urbanization, economic urbanization, population urbanization, spatial urbanization, and green urbanization (see Table 1). The new urbanization index was calculated using the entropy weight method and served as a measurement indicator for the level of new urbanization.

**Table 1:** New urbanization indicator system.

Target layer	Criterion layer	Primary indicators	Secondary indicators	Expected changes
New urbanization	Social urbanization	Informatization level	The number of internet users	+
		Logistics supply and demand situation	Total road freight volume	+
		Public service level	Total highway passenger volume	+
	Economic urbanization	Economic scale level	GDP/total population	+
		Economic efficiency level	GDP/fixed assets investment	+
		Urban industrial structure	The output values of the second and third industries/total output value	+
	Population urbanization	Population urbanization rate	Urban population/total population	+
		Proportion of urban employment	The sum of employees in the second and third industries/total employees	+
		Urban population density	Population density	+
	Spatial urbanization	Land urbanization rate	Urban built-up area/total area	+
		Land carrying capacity	Urban built-up area/total population	+
		Land development level	Investment in real estate development	+
	Green urbanization	Urban livability level	Green coverage rate in urban built-up areas	+
		Industrial pollution treatment	Comprehensive utilization rate of industrial solid waste	+
		Domestic pollution treatment	Domestic sewage treatment rate	+

#### Core explanatory variable

Financial development level (*FD*): Based on the approach of Shahbaz et al. [30], this study used the proportion of deposit and loan balances to GDP (*FD*_*1*_) as a measure of financial development level. The proportion of loan balance in GDP (*FD*_*2*_) was used as a substitute indicator of financial development level for the robustness test.

#### Mediating variables

(1) Infrastructure investment (*II*): The total amount of urban fixed assets investment was used to measure infrastructure investment [31], and it was treated logarithmically. (2) Human capital level (*HC*): The number of students in colleges and universities was used to measure the human capital level and subjected logarithmic processing [32]. (3) Industrial structure optimization (*ISO*): *ISO* was expressed using a weighted representation of industrial structure upgrading (*ISU*) and industrial structure rationalization (*ISR*), with a weight ratio of *ISU*:*ISR* of 2:3 [33]. *ISU* was measured by the ratio of the output value of the tertiary industry to the output value of the secondary industry, while *ISR* was calculated using the Thiel index. The formula is as follows:

$$TL=\sum_{i=1}^{n}\left(\frac{Y_{i}}{Y}\right)\;\text{ln}\;\left(\frac{Y_{i}}{L_{i}}/\frac{Y}{L}\right),$$ (6)

where *Y*_*i*_ and *L*_*i*_ represent the output value of the *i-industry* and the number of employees, respectively (*i* = 1, 2, 3), *Y* represents the total output value, and *L* represents the total number of employees.

#### Control variables

(1) Macro-control ability (*MC*): The proportion of general budget expenditure in GDP was used to measure macro-control ability. (2) Commercial service activity level (*CS*): The proportion of leasing and business service employees in the total population was used to measure the level of commercial service activity. (3) Technology research and development level (*TR*): The proportion of the number of people employed in scientific research, technical services, and geological surveys in the total population was used to measure the level of technology research and development.

### Data sources

In this paper, the 2003–2019 data of 108 cities at or above the prefecture level in China’s Yangtze River Economic Belt were taken as research samples, and Tongren and Bijie, which have serious data losses, were excluded. Considering that a large number of administrative divisions of prefecture-level cities were adjusted before 2003, to ensure the comparability and continuity of data, this study selected the period from 2003 to 2019 as the research interval. The data in this paper were mainly derived from the China City Statistical Yearbook, provincial and municipal Statistical Yearbook, and the Statistical Bulletin of National Economic and Social Development for 2004–2020. Individual missing data were supplemented by the interpolation method, and the descriptive statistics of each variable are shown in Table 2.

**Table 2:** Descriptive statistics of variables.

Variables	Symbols	Observations	Mean	Std. Dev	Minimum	Maximum
New urbanization	*NU*	1,836	0.353	0.083	0.148	0.792
Financial development level	*FD*_*1*_	1,836	2.143	0.891	0.764	6.434
	*FD*_*2*_	1,836	0.862	0.453	0.260	3.480
Infrastructure investment	*II*	1,836	6.492	1.261	3.086	9.890
Industrial structure optimization	*ISO*	1,836	0.530	0.197	0.141	2.172
Human capital level	*HC*	1,836	1.210	1.534	-6.908	4.612
Macro-control ability	*MC*	1,836	0.168	0.083	0.049	0.688
Commercial service activity level	*CS*	1,836	0.199	0.366	0.002	5.200
Technology research and development level	*TR*	1,836	0.169	0.235	0.012	2.465

## Empirical results analysis

### Benchmark model regression results

Based on Model 1, benchmark regression analysis was conducted, and the results are shown in Table 3. Column (1) shows only estimates of the impact of financial development level *FD*_*1*_ on the level of new urbanization, while columns (2) to (4) show the results of gradually adding the control variables, such as *MC*, *CS*, and *TR*. The results show that when no control variables were added, the coefficient of financial development was significantly positive at a confidence level of 1%, indicating that financial development can promote the construction of new urbanization. When the control variables were gradually added, the coefficient of financial development was still significantly positive at the 1% significance level, with only a change in coefficient size, indicating that the result was robust. The robustness test was re-performed using *FD*_*2*_ *to* replace *FD*_*1*_ of column (4), and the results are shown in column (5). The coefficient of financial development level remained significantly positive at the 1% confidence level, which further indicates that the benchmark regression results were robust and that financial development could indeed promote the construction of new urbanization. Hypothesis 1 is tested. This is because it is difficult to support the new urbanization construction solely through financial input the new urbanization construction, while financial development provides financial support for promoting the new urbanization construction [34].

**Table 3:** Benchmark regression results.

Variables	(1)	(2)	(3)	(4)	(5)
***FD***_***1***_	0.068*** (31.06)	0.040*** (16.05)	0.031*** (12.39)	0.030*** (12.48)	-
***FD***_***2***_	-	-	-	-	0.046*** (12.90)
***MC***	-	0.373*** (19.54)	0.383*** (20.84)	0.371*** (21.19)	0.432*** (28.58)
***CS***	-	-	0.041*** (11.78)	-0.002 (-0.52)	-0.001 (-0.18)
***TR***	-	-	-	0.145*** (13.60)	0.146*** (13.78)
**Constant**	0.207*** (43.26)	0.206*** (47.52)	0.215*** (50.70)	0.204*** (49.57)	0.217*** (65.10)
**Urban FE**	Yes	Yes	Yes	Yes	Yes
**Adj. R** ^**2**^	0.318	0.441	0.483	0.533	0.535
**F-value**	964.9***	779.7***	607.6***	550.5***	556.1***
**Observations**	1,836	1,836	1,836	1,836	1,836

Note:

*, **, and *** indicate passing the test at the 10%, 5%, and 1% significance levels, respectively. The values in parentheses represent the t-values corresponding to the estimated coefficients, the same below.

### Mediation effect test

To further study the mechanism of financial development on new urbanization, this study tested the mediation effect from three aspects: infrastructure investment, industrial structure optimization, and human capital enhancement. The test results are shown in Table 4.

**Table 4:** Test results of the mediation effect.

Variables	*II*	*ISO*	*HC*	*NU*
	(1)	(2)	(3)	(4)
***FD***_***1***_	0.510*** (10.56)	0.098*** (12.39)	0.218*** (5.11)	0.012*** (6.07)
***II***	-	-	-	0.030*** (30.95)
***ISO***	-	-	-	0.017*** (3.04)
***HC***	-	-	-	0.004*** (3.52)
**Control variable**	Yes	Yes	Yes	Yes
**Constant**	2.899*** (34.67)	0.358*** (26.17)	-0.231*** (-3.13)	0.111*** (24.36)
**Urban FE**	Yes	Yes	Yes	Yes
**Adj. R** ^**2**^	0.644	0.112	0.245	0.729
**F-value**	858.9***	85.79***	176.8***	719.7***
**Observations**	1,836	1,836	1,836	1,836

According to the single mediation effect analysis, the mediation effect of infrastructure investment as an intermediary variable was 0.015 (= 0.510 × 0.030) and at a significance level of 1%. This indicates that financial development can promote the construction of new urbanization through infrastructure investment channels [35]. The mediation effect of industrial structure optimization as an intermediary variable was 0.002 (= 0.098 × 0.017), which was significant at the 1% level, indicating that financial development had a positive impact on the construction of new urbanization by improving the level of the industrial structure optimization, that is, promoting the improvement of the level of new urbanization [36]. The mediation effect of human capital enhancement as an intermediary variable was 0.001 (= 0.218 × 0.004), which was significant at a significance level of 1%, indicating that financial development further improved the level of new urbanization by promoting the improvement of human capital. Based on the above analysis, it can be concluded that financial development can indirectly promote the construction of new urbanization through three channels: infrastructure investment, industrial structure optimization, and human capital enhancement [37]. Hypothesis 2 is tested.

Based on the total mediation effect analysis, the total mediation effect was 0.018 (= 0.002 + 0.015 + 0.001), which indicates that financial development had a positive effect on new urbanization through three channels: infrastructure investment, industrial structure optimization, and human capital enhancement. According to the regression results of column (4), the direct effect of financial development on new urbanization was 0.012, which was significant at the 1% significance level. This shows that financial development itself was also conducive to improving the level of new urbanization after excluding the influence of three intermediary variables: infrastructure investment, industrial structure optimization, and human capital improvement. The regression coefficient symbols of indirect effect and direct effect were the same, indicating that there was partial mediation effect in this model, and indirect effect accounted for 60% (= 0.018/0.030) of the total effect.

Following the outcomes above, a comparison of the single mediation effect revealed that the mediating effects of infrastructure investment, industrial structure optimization, and human capital enhancement on new urbanization were 0.015, 0.002, and 0.001, respectively, accounting for 83%, 11%, and 6% of the total mediating effects, respectively. Further, the mediation effect of the infrastructure investment channel was significantly higher than that of industrial structure optimization and the human capital enhancement channel, and its proportion in the total intermediary effect was also significantly higher than the latter two channels, indicating that financial development mainly promoted new urbanization construction through infrastructure investment. This may be because infrastructure construction has a wide range, large capital demand, and is the foundation of new urbanization construction, while financial development can only provide the capital demand for infrastructure investment, and thus produce a stronger intermediary effect [38].

### Robustness test

To further verify the robustness and reliability of the above empirical results, this study chose to replace key variables and divide the sample.

#### Replace key variable

We selected the proportion of loan balance to GDP (*FD*_*2*_) to measure the level of financial development and conducted multiple intermediary tests based on Eqs 2–5. The test results are shown in Table 5.

**Table 5:** Robustness test results.

Variables	*II*	*ISO*	*HC*	*NU*
	(1)	(2)	(3)	(4)
***FD***_***2***_	0.783*** (10.78)	0.147*** (12.35)	0.201*** (3.11)	0.019*** (6.66)
***II***	-	-	-	0.030*** (30.59)
***ISO***	-	-	-	0.016*** (2.94)
***HC***	-	-	-	0.004*** (3.88)
**Control variable**	Yes	Yes	Yes	Yes
**Constant**	3.127*** (46.10)	0.404*** (36.33)	-0.057 (-0.94)	0.117*** (26.63)
**Urban FE**	Yes	Yes	Yes	Yes
**Adj. R** ^**2**^	0.645	0.112	0.238	0.730
**F-value**	862.2***	85.57***	171.1***	723.8***
**Observations**	1,836	1,836	1,836	1,836

A comparative analysis of results in Tables 4 and 5 shows that although financial development was measured in different ways, the estimated coefficients in each model did not change much, and the symbols of the estimated coefficients did not change. Table 5 also shows that the total intermediary effect was 0.027 (= 0.046–0.019), accounting for 59% (= 0.027/0.046) of the total effect. The mediation effect of infrastructure investment as an intermediary variable was 0.023 (= 0.783 × 0.030), accounting for 85% (= 0.023/0.027) of the total mediation effect, which was significantly larger than the other two mediation variables. This outcome is consistent with the analysis results obtained in Table 4, indicating that the empirical estimation results in this study were robust and reliable.

#### Results of sample intervals

According to the three batches of national comprehensive pilot lists for new urbanization released by the National Development and Reform Commission of China in February 2015, November 2015, and December 2016, the 108 cities in the Yangtze River Economic Belt were divided into 68 pilot cities and 40 non-pilot cities to study the relationship between financial development and new urbanization. The research results are shown in Tables 6 and 7. The division of pilot cities and non-pilot cities was conducted as follows: (1) prefecture-level cities in Jiangsu and Anhui, the national comprehensive pilot areas of new urbanization, were classified as pilot cities; (2) prefecture-level cities in the pilot list were classified as pilot cities; (3) certain areas within the jurisdiction of a prefecture-level city included in the pilot list were classified as pilot cities; and (4) the remaining cities were considered as non-pilot cities.

**Table 6:** Benchmark regression and mediation effect test of pilot cities.

Variables	Benchmark regression	Infrastructure investment	Industrial structure optimization	human capital enhancement	New urbanization
	*NU*	*II*	*ISO*	*HC*	*NU*
	(1)	(2)	(3)	(4)	(5)
***FD***_***1***_	0.016*** (5.57)	0.245*** (4.41)	0.112*** (12.13)	0.111*** (2.65)	0.007*** (2.79)
***II***	-	-	-	-	0.030*** (21.83)
***ISO***	-	-	-	-	0.014* (1.68)
***HC***	-	-	-	-	0.003* (1.82)
**Control variable**	Yes	Yes	Yes	Yes	Yes
**Constant**	0.220*** (45.15)	3.166*** (34.33)	0.355*** (23.26)	-0.003 (-0.04)	0.119*** (19.77)
**Urban FE**	Yes	Yes	Yes	Yes	Yes
**Adj. R** ^**2**^	0.613	0.729	0.220	0.408	0.752
**F-value**	475.8	793.2	99.19	216.5	510.3
**Observations**	1,156	1,156	1,156	1,156	1,156

**Table 7:** Benchmark regression and mediation effect test in non-pilot cities.

Variables	Benchmark regression	Infrastructure investment	Industrial structure optimization	human capital enhancement	New urbanization
	*NU*	*II*	*ISO*	*HC*	*NU*
	(1)	(2)	(3)	(4)	(5)
***FD***_***1***_	0.043*** (11.13)	0.718*** (8.38)	0.108*** (7.65)	0.298*** (3.33)	0.020*** (6.27)
***II***	-	-	-	-	0.028*** (19.63)
***ISO***	-	-	-	-	0.018** (2.26)
***HC***	-	-	-	-	0.004*** (3.11)
**control variable**	Yes	Yes	Yes	Yes	Yes
**Constant**	0.179*** (25.65)	2.526*** (16.38)	0.343*** (13.50)	0.554*** (-3.44)	0.103*** (14.86)
**Urban FE**	Yes	Yes	Yes	Yes	Yes
**Adj. R** ^**2**^	0.459	0.564	0.0674	0.132	0.706
**F-value**	154.6	230.2	23.02	36.49	240.0
**Observations**	680	680	680	680	680

According to the benchmark regression results in Tables 6 and 7, the financial development of both pilot cities and non-pilot cities significantly improved the level of new urbanization. According to the results of the intermediary effect test, financial development guided infrastructure investment, promoted the optimization of industrial structure, and accelerated the enhancement of human capital, thus promoting the construction of new urbanization. Further, the intermediation effect of infrastructure investment in pilot cities accounted for 82% of the total intermediation effect, while that of infrastructure investment in non-pilot cities accounted for 87% of the total intermediation effect. This indicates that both pilot cities and non-pilot cities indirectly promoted new urbanization construction mainly through infrastructure investment channels, which further verifies the robustness of the regression results.

### Regional heterogeneity test

To further investigate the impact of financial development on the regional heterogeneity of new urbanization, this study conducted benchmark regression and mediation effect tests in downstream cities and middle-upstream cities of the Yangtze River Economic Belt. The regression results are shown in Tables 8 and 9.

**Table 8:** Benchmark regression and mediation effect test in downstream cities of the Yangtze River Economic Belt.

Variables	Benchmark regression	Infrastructure investment	Industrial structure optimization	human capital enhancement	New urbanization
	*NU*	*II*	*ISO*	*HC*	*NU*
	(1)	(2)	(3)	(4)	(5)
***FD***_***1***_	0.024*** (7.17)	0.387*** (6.00)	0.113*** (10.64)	0.084** (2.41)	0.009*** (3.07)
***II***	-	-	-	-	0.023*** (11.67)
***ISO***	-	-	-	-	0.040*** (4.03)
***HC***	-	-	-	-	0.021*** (5.72)
**control variable**	Yes	Yes	Yes	Yes	Yes
**Constant**	0.248*** (43.06)	3.660*** (32.74)	0.249*** (13.53)	0.592*** (9.79)	0.141*** (18.62)
**Urban FE**	Yes	Yes	Yes	Yes	Yes
**Adj. R** ^**2**^	0.654	0.700	0.342	0.457	0.792
**F-value**	339.3***	416.7***	101.6***	157.3***	385.2***
**Observations**	697	697	697	697	697

**Table 9:** Benchmark regression and mediation effect test in middle-upstream cities of the Yangtze River Economic Belt.

Variables	Benchmark regression	Infrastructure investment	Industrial structure optimization	human capital enhancement	New urbanization
	*NU*	*II*	*ISO*	*HC*	*NU*
	(1)	(2)	(3)	(4)	(5)
***FD***_***1***_	0.031*** (9.35)	0.553*** (8.12)	0.093*** (8.25)	0.336*** (5.00)	0.012*** (4.53)
***II***	-	-	-	-	0.031*** (26.36)
***ISO***	-	-	-	-	0.010 (1.44)
***HC***	-	-	-	-	0.003** (2.21)
**control variable**	Yes	Yes	Yes	Yes	Yes
**Constant**	0.177*** (31.36)	2.404*** (20.92)	0.431*** (22.72)	-0.775*** (-6.84)	0.099*** (16.74)
**Urban FE**	Yes	Yes	Yes	Yes	Yes
**Adj. R** ^**2**^	0.470	0.636	0.0174	0.222	0.703
**F-value**	270.1***	515.0***	22.54***	98.73***	396.0***
**Observations**	1,139	1,139	1,139	1,139	1,139

According to the benchmark regression results in Tables 8 and 9, the coefficients of the financial development in downstream cities and middle-upstream cities were significantly positive at the 1% significance level, with only differences in coefficient sizes. Specifically, the estimated coefficient for financial development in downstream cities was 0.024, while the regression coefficient for financial development in middle-upstream cities was 0.031. The results indicate that the financial development of middle-upstream cities had a stronger promoting effect on new urbanization. This may be because the level of new urbanization in downstream cities had reached a relatively high level, while the new urbanization level of middle-upstream cities still had a large room for progress [8,39]. Therefore, the marginal effect of the financial development of middle-upstream cities on the new urbanization was relatively large.

The regression results of the mediation effect in Tables 8 and 9 show that there were obvious differences in the effect of three intermediary variables in the process of financial development affecting new urbanization between downstream cities and middle-upstream cities. Specifically, the mediation effect of infrastructure investment, industrial structure optimization, and human capital enhancement in downstream cities accounted for 59%, 30%, and 11% of the total mediation effect, respectively, indicating that the financial development of downstream cities mainly promoted the construction of new urbanization through infrastructure investment and industrial structure optimization. The mediation effect of infrastructure investment, human capital enhancement, and industrial structure optimization in middle-upstream cities accounted for 90%, 5.2%, and 4.8% of the total mediation effect, respectively, indicating that the financial development of middle-upstream cities mainly depended on infrastructure investment channels to indirectly promote the construction of new urbanization [11,40]. A comparison of the effects of various mediating variables on downstream cities and middle-upstream cities revealed that the effect of infrastructure investment in both regions was relatively large, while the effect of human capital enhancement was relatively small. Therefore, the different leading factors of downstream cities and upstream cities were primarily from the different degrees of influence of industrial structure optimization on new urbanization in the two regions. According to the regression results of column (5) in Tables 8 and 9, the effect of industrial structure optimization of downstream cities on new urbanization was significantly higher than that of middle-upstream cities. The reason may be that the strategic emerging industries of the downstream cities were developed and the industrial structure was mature, which was conducive to promoting the construction of new urbanization. However, some middle-upstream cities still had industries with excess capacity as their local pillar industries, the strategic emerging industries were relatively few, and the industrial value chain was low end; thus, the disconnection between industrial development and the process of urbanization could have easily led to low urbanization efficiency [41].

## Conclusion and policy recommendations

Based on panel data of 108 cities in China’s Yangtze River Economic Belt from 2003 to 2019, this study used a multiple mediation model to study the mechanism of financial development on new urbanization. The main conclusions are as follows: (1) Financial development plays a significant positive role in promoting the level of new urbanization. Financial development can indirectly promote the construction of new urbanization by guiding infrastructure investment, promoting the optimization of industrial structure, and accelerating the enhancement of human capital. (2) Compared with downstream cities, the financial development of middle-upstream cities plays a stronger role in promoting new urbanization. Further, the financial development of downstream cities mainly promotes new urbanization construction through infrastructure investment and industrial structure optimization, while middle-upstream cities are more dependent on infrastructure investment channels.

Based on the above conclusions, the following two policies are suggested:

1. The National New–type Urbanization Plan (2014–2020) in China pointed out that the new urbanization construction has increased the financial pressure of local governments, and infrastructure, industrial structure, and human capital play an important role in the new urbanization construction, but ignored the importance of financial development in its construction. Therefore, the government should expand investment and financing channels and improve the level of financial development to promote the construction of new urbanization. They should also increase financial support for infrastructure investment, industrial structure optimization, and human capital enhancement to further promote the construction of new urbanization. To this end, first, the government should reasonably guide private capital to invest in urban infrastructure construction, thereby effectively filling the financial gap of the financial sector in infrastructure construction with the help of financial support from the private sector. The government should assist in new infrastructure construction to accelerate the construction of new urbanization. Second, the city’s dominant and leading industries should receive active support to promote the continuous upgrading of industries. Further, full play should be given to the role of financial resource allocation in promoting the coordinated development of various industries, thus achieving the rationalization of industrial structure. Third, the concept of education should be deeply implemented to increase investment in local talent training. improve the housing security system to provide more financial support for pensions, medical care, fertility, environment, culture, and other public services, and thus improve the introduction of talent.
2. The Outline of Yangtze River Economic Belt Development in China calls for strengthening new urbanization, but there is no differentiated support policy based on the difference between downstream cities and middle-upstream cities. Regional differences in the character of new urbanization in the Yangtze River Economic Belt are substantial. Differentiated urbanization development models should be adopted by adhering to locational and category-specific principles [42]. Therefore, the government should implement differentiated support policies according to local conditions. Given that middle-upstream cities play a stronger role in promoting new urbanization than downstream cities, the government should increase the financial support of middle-upstream cities and deepen the structural reform of the financial supply side to promote the coordinated development of new urbanization among cities. However, both downstream and middle-upstream cities should increase investment in infrastructure construction to promote the construction of new urbanization. Also, downstream cities should focus on providing financial support for the optimization of industrial structure.

## Supporting information

S1 File Dataset. (XLSX)
